# Interrelationships Within the Bacterial Flora of the Female Genital Tract

**DOI:** 10.1155/S1064744997000525

**Published:** 1997

**Authors:** Henry J. Carson, Paul G. Lapoint, Gilles R. G. Monif

**Affiliations:** 1 Department of Pathology Resurrection Medical Center Chicago IL USA; 2 Department of Obstetrics and Gynecology Creighton University School of Medicine 601 N. 30th Street Omaha NE 68131 USA

## Abstract

Analysis of 240 consecutive vaginal swabs using the compatibility profile technique revealed that only 2 bacteria have the ability to be a sole isolate and as such a candidate to be a major aerobic regulator of the bacterial flora of the female genital tract (BFFGT). Compatibility profiles of *Lactobacillus* and *Gardnerella vaginalis* have shown that these organisms shared compatibility profiling for the majority of the normal bacterial constituents of the female genital tract. Dominance disruption appears to come from the addition of compatible co-isolates and presumed loss of numerical superiority. These phenomena appear to be the keys to reregulation of BFFGT. *Lactobacillus* appears to be the major regulator of both *G. vaginalis* and anaerobic bacteria. When additional organisms are added to the bacterial flora, they may add to or partially negate the inhibitory influence of *Lactobacillus* on the BFFGT. Inhibitor interrelationships appear to exist between coagulase-negative staphylococci and *Staphylococcus aureus* and the group B streptococci (GBS) and other beta hemolytic streptococci. Facilitating interrelationships appear to exist between *S. aureus* and the GBS and selected *Enterobacteriaceae*.


Infectious Diseases in Obstetrics and Gynecology 5:303–309 (1997)
© 1998 Wiley-Liss, Inc.

### Interrelationships Within the Bacterial Flora of the Female Genital Tract

**Henry J. Carson,[1]* Paul G. Lapoint,[2] and Gilles R.G. Monif[2]**
[1]*Department of Pathology, Resurrection Medical Center, Chicago, IL*
[2]*Department of Obstetrics and Gynecology, Creighton University School of Medicine, Omaha, NE*

ABSTRACT

**Analysis of 240 consecutive vaginal swabs using the compatibility profile technique revealed that only 2 bacteria have the ability to be a sole isolate and as such a candidate to be a major aerobic regulator of the bacterial flora of the female genital tract (BFFGT). Compatibility profiles of *Lactobacillus* and *Gardnerella vaginalis* have shown that these organisms shared compatibility profiling for the majority of the normal bacterial constituents of the female genital tract. Dominance disruption appears to come from the addition of compatible co-isolates and presumed loss of numerical superiority. These phenomena appear to be the keys to reregulation of BFFGT. *Lactobacillus* appears to be the major regulator of both *G. vaginalis* and anaerobic bacteria. When additional organisms are added to the bacterial flora, they may add to or partially negate the inhibitory influence of *Lactobacillus* on the BFFGT. Inhibitor interrelationships appear to exist between coagulase-negative staphylococci and *Staphylococcus aureus* and the group B streptococci (GBS) and other beta hemolytic streptococci. Facilitating interrelationships appear to exist between *S. aureus* and the GBS and selected *Enterobacteriaceae*. Infect Dis. Obstet. Gynecol. 5:303–309, 1997.** © 1998 Wiley-Liss, Inc.

KEY WORDS
bacterial flora; female genital tract; bacterial interrelationships; *Lactobacillus; Gardnerella vaginalis*

The female genital tract microbiology is postulated to be a tightly orchestrated, dynamic system which follows defined patterns of regulation.[1,2] Most vaginal cultures have 3–6 bacteria.[3,4] Only a minority of cultures have a single bacterium. When group B *Streptococcus* (GBS) is present in high numbers (>$10^7$/cfu/g of vaginal fluid), the concomitant bacterial flora tends to be simplified (Monif, unpublished data). When present in the vaginal flora at <$10^6$/cfu/g of vaginal fluid, co-isolation of multiple bacteria with numerical superiority by one or more of these co-isolates can be demonstrated. Perception of a high degree of governance has been obscured by the various combinations of bacterial combinations which can function within the limits imposed by a given microbiological environment. Our perception of bacterial dominance emanates from studies with the GBS which implied that some form of bacterial interference selectively functions governance of that particular organism.[5] The composition of each bacterial vaginal flora appears to be tightly regulated until there is an alteration of the microbiological environment or one or more of the governing members of the microbiological flora is removed.[1,7] When antibiotics are administered, a regulatory interrelationship is disrupted which creates a void into which existing microbial organisms can expand to governance or new microbial organisms can move into the void and potentially alter the quantitative interrelationship of those bacteria present.

Bacterial studies of the bacterial flora of the fe-

*Correspondence to: Dr. Henry J. Carson, c/o Gilles R.G. Monif, M.D., Department of Obstetrics and Gynecology, Creighton University School of Medicine, 601 N. 30th Street, Omaha, Nebraska 68131.

Received 20 January 1997
Accepted 25 September 1997

Clinical Study male genital tract (BFFGT) have rarely analyzed the interreaction of the various constituents to each other. Most of the information concerning governance is based on *in vitro* work which analyzed the presence or absence of bacterial interference. For *in vitro* data to have *in vivo* relevance, the quantitative representation of the target bacteria must be assured. The ability of one bacterium to adversely affect the replication of other bacteria has been well documented, and is presumed to be a principal mechanism accounting for the constituency of the BFFGT.

### MATERIALS AND METHODS

### Specimen Handling

All specimens received over a 6 month period were cultured for aerobic and anaerobic bacteria and *Candida* species. These specimens were received primarily from ambulatory care clinics (Table 1). The specimens had been obtained with culturette II swabs. All cultures submitted for cervicitis or vaginal discharge or pruritus and those from pregnant women were analyzed separately. Gram stains were performed on all cases, and the swabs were cultured for aerobes on colistin-nalidixic acid agar, chocolate blood agar, and eosin methylene-blue agar, and for anaerobes on phenylethyl alcohol agar, Centers for Disease Control (CDC) and anaerobic blood agar, kanamycin-vancomycin agar, and enriched thioglycolate medium (media were obtained from BBL, Becton Dickinson Microbiology Systems, Cockeysville, MD). Aerobic media were incubated at 35°C and read after 24 h, while anaerobic media were incubated in 70% nitrogen and 30% carbon dioxide at 35°C and read after 48 h.

Additional workup of aerobic cultures involved interpretation of Gram stains, hemolysis patterns, and colony characteristics. These findings plus catalase (using 0.1% hydrogen peroxide) and coagulase (Staphaurex, Murex Diagnostics, Darford, England) test results were used in the Vitek System (bioMerieux, Hazelwood, MO) for identification and antibiotic sensitivities.

Additional workup of anaerobic cultures required confirmation of anaerobic bacteria by inoculating the suspected anaerobic organism onto blood agar and anaerobic CDC media. After overnight incubation in aerobic and anaerobic conditions, respectively, organisms which grew on the latter medium but not the former were worked up as anaerobes. Gram stains, colony morphology, and the Rapid ANA II system (Innovative Diagnostic Systems, Inc., Norcross, GA) contributed to the identification of these organisms.

TABLE 1. Specimens cultured for the bacterial studies

| Indication | No. of specimens derived from women in a given category |
|---|---|
| Abdominal pain | 75 |
| Pregnancy (rule out GBS) | 67 |
| Sexually transmitted disease evaluation | 19 |
| Cervicitis/vaginal discharge | 23 |
| No diagnosis | 30 |
| Pelvic pain | 7 |
| Fever/sepsis | 5 |
| Hemorrhage | 6 |
| Rape | 5 |
| Urinary tract infection | 5 |
| Abortion | 4 |
| Other | 5 |
| Total | 240 |

### Comparative Profiling Technique

The microbiological data were analyzed by the comparative profiling technique developed by G.R.G.M. The technique starts by identifying those bacteria which occurred as a single isolate and then looks at the co-isolates present when only 2 bacteria are recovered. The most prevalent of these bacteria is then added to the initial target bacteria and co-isolates are identified when only 3 bacteria are recovered. This process is again repeated using cultures when 4 bacteria are present, etc. This process of additive bacterial grouping lends to the establishment of a compatibility profile. By inference those bacteria which are not present may be susceptible to bacterial interference by one of the target organisms or its subsequent additive isolates.

### Statistical Analysis

The microbiological data were analyzed statistically using the standard or enhanced association Student's t-test for evidence of bacterial interference. Analysis of bacterial interrelationships was restricted to those bacteria isolated in sufficient prevalence so that statistical validity could be achieved. Where appropriate (sample size $<5$) Fisher's exact test was used. $P < 0.05$ was considered to be significant.

**TABLE 2. Bacterial isolates**

| Isolates | No. |
|---|---|
| Aerobic | |
| Lactobacilli | 113 |
| Diphtheroids | 33 |
| *Gardnerella vaginalis* | 96 |
| Coagulase-negative *Staphylococcus* | 132 |
| *S. aureus* | 11 |
| *S. saprophyticus* | 1 |
| Group D *Enterococcus* | 96 |
| Alphahemolytic streptococci | 26 |
| Gammahemolytic streptococci | 17 |
| GBS | 49 |
| Group C *Streptococcus* | 1 |
| Group D *Streptococcus* | 5 |
| Group F *Streptococcus* | 2 |
| *Escherichia coli* | 58 |
| *Klebsiella pneumoniae* | 10 |
| *K. oxytoca* | 2 |
| *Proteus mirabilis* | 6 |
| *Enterobacteriae cloacae* | 2 |
| *Haemophilus influenzae* | 1 |
| *H. parainfluenzae* | 3 |
| *Acinetobacter* species | 2 |
| *Neisseria sicca* | 1 |
| *N. gonorrheae* | |
| Anaerobic | 1 |
| *Clostridium* | 3 |
| *Propionbacterium* | 1 |
| *Bifidobacterium* | 1 |
| *Fusobacterium* | 2 |
| *Mobilincus* | 1 |
| *Peptostreptococcus* | 29 |
| *P. anaerobius* (7) | |
| *P. tetradiens* (1) | |
| *P. magnus* (6) | |
| *P. asaccharolyticus* (2) | |
| Unspeciated (13) | |
| Gram-positive anaerobic bacilli | 5 |
| *Prevotella bivia* | 25 |
| *B. melaninogenica* (5) | |
| Unspeciated (1) | |
| *Bacteroides* | 30 |
| *B. caccae* (1) | |
| *B. corporis* (4) | |
| *B. disiens* (3) | |
| *B. fragilis* (8) | |
| *B. intermedius* (2) | |
| *B. uniformis* (2) | |
| *B. vulgatus* (1) | |
| Unspeciated (8) | |
| Gram-negative anaerobic bacilli | 7 |

### RESULTS

Seven hundred eighty of the isolates were achieved: 783 aerobic isolates and 104 anaerobic isolates. The overall tabulation of bacterial isolates is listed in Table 2. From the microbiological data, the cultures and bacterial specimen handling techniques were adequate for aerobic bacteria; however, the low prevalence of gram-positive anaerobic rod (anaerobic lactobacilli) *Eubacterium, Propionbacterium, Bifidobacterium,* and *Clostridium* was consistent with the concept that the anaerobic data were probably more reflective of high multiplicity replication rather than identification of true prevalence.

### Comparative Profiles

When comparative profiles were done using the 781 isolates achieved from 239 vaginal cultures, the only bacteria which achieved a single isolate status were *Lactobacillus* and *Gardnerella vaginalis* (see Tables 3, 5). The compatibility profiles were constructed based on these two bacteria. As would be anticipated, the recovery of *Lactobacillus* was at the lower end of recorded prevalence and that for *G. vaginalis* was at the upper end of their recorded prevalence in normal women without overt diseases.

### *Lactobacillus*

*Lactobacillus* was identified in 131 vaginal cultures (Table 3). In 7 cultures, *Lactobacillus* was the sole bacteria isolate. On the first level with 2 isolates per culture, compatibility profiling co-isolates were coagulase-negative *Staphylococcus* (9), group D *Enterococcus* (8), aerobic gram-positive bacilli (3), *Enterobacteriaceae* (2), and alpha hemolytic *Streptococcus* (1). On level two with the combination of *Lactobacillus* and coagulase-negative *Staphylococcus* used to identify the third isolate within the cultures with just 3 isolates, the results were as follows: group D *Enterococcus* (8), *Enterobacteriaceae* (3), alpha or gamma *Streptococcus* (3), aerobic gram-positive bacilli (1), and group D *Streptococcus* (1). When the combination of *Lactobacillus* and group D *Enterococcus* was used to identify the third isolate within cultures with only 3 isolates, the results were as follows: coagulase-negative *Staphylococcus* (8), GBS (2), *Enterobacteriaceae* (1), alpha *Streptococcus* (1), and anaerobic bacteria (1). On level three when the combination of *Lactobacillus,* coagulase-negative *Staphylococcus,* and group D *Enterococcus* was used to identify bacteria present, the following co-isolates were identified: *Enterobacteriaceae* (4), GBS (2), anaerobes (3), diphtheroids (1), aerobic gram-positive bacilli (1), and alpha or gamma *Streptococcus* (1). When *Lactobacillus* was present, *G. vaginalis* was a concomitant isolate in 7 of 108 cultures. When no anaerobes were present, only one isolate

TABLE 3. Compatibility profile: *Lactobacillus*

| | | |
|---|---|---|
| No. of cultures containing one or more species of *Lactobacillus* | | 113/240 |
| No. of cultures in which *Lactobacillus* was the sole isolate | | 7/113 |
| No. of cultures in which a single co-isolate was achieved with *Lactobacillus* | | 24/106 |
| Coagulase-negative *Staphylococcus* | 9 | |
| *Enterococcus* | 8 | |
| Anaerobic gram-positive bacilli | 3 | |
| *Enterobacteriaceae* | 2 | |
| Alpha hemolytic *Streptococcus* | 1 | |
| Gamma hemolytic *Streptococcus* | 1 | |
| No. of cultures in which co-isolates with *Lactobacillus* and coagulase-negative *Staphylococcus* | | 16 |
| *Enterococcus* species | 8 | |
| *Enterobacteriaceae* | 3 | |
| Alpha or gamma staphylococci | 3 | |
| Aerobic gram-positive bacilli | 1 | |
| Group D *Streptococcus* | 1 | |
| No. of cultures in which co-isolates with *Lactobacillus* and group D *Enterococcus* | | 13 |
| Coagulase-negative *Staphylococcus* | 8 | |
| GBS | 2 | |
| *Enterobacteriaceae* | 1 | |
| Alpha *Streptococcus* | 1 | |
| Anaerobic bacteria | 1 | |
| No. of cultures in which a co-isolate is identified with *Lactobacillus*, coagulase-negative *Staphylococcus*, and *Enterococcus* | | 12 |
| *Enterobacteriaceae* | 4 | |
| GBS | 2 | |
| Anaerobes | 3 | |
| Diphtheroids | 1 | |
| Anaerobic gram-positive bacilli | 1 | |
| Gamma hemolytic *Streptococcus* | 1 | |

of *G. vaginalis* was recorded (Table 4). When *G. vaginalis* was recovered with *Lactobacillus*, it occurred in 6 of the 23 cultures in which both *Lactobacillus* and anaerobic bacteria coexisted. If *Lactobacillus* is present, the probability of recovering *G. vaginalis* is statistically reduced ($P > 0.0001$).

### *G. vaginalis*

*G. vaginalis* was identified in 96 cultures (Table 5). In eight cultures, *G. vaginalis* was the sole isolate achieved. On the first level with 2 isolates per culture, the compatibility profiling identified 16 co-isolates: coagulase-negative group D *Enterococcus* (5), aerobic gram-positive bacilli (2), alpha and gamma GBS (2), and diphtheroids (1). On level two with cultures containing *G. vaginalis* and group D *Enterococcus* within cultures with 3 isolates, the compatibility isolates in 6 was *Escherichia coli* (4), GBS (1), anaerobes (1), diphtheroids (1), and alpha *Streptococcus* (1). On the second level with 3 iso-

TABLE 4. Interrelationships of *Lactobacillus* and *G. vaginalis*

| | |
|---|---|
| No. of cultures in which *Lactobacillus* and *G. vaginalis* were co-isolates | 7/113 |
| No. of cultures in which *Lactobacillus* and *G. vaginalis* were co-isolates in the absence of anaerobic isolates | 1/113 |

TABLE 5. Compatibility profile: *Gardnerella*

| | | |
|---|---|---|
| No. of cultures in which *G. vaginalis* was identified | | 96/240 |
| No. of cultures in which *G. vaginalis* was the sole isolate | | 8/96 |
| No. of cultures in which a single co-isolate was achieved with *G. vaginalis* | | 16/88 |
| Coagulase-negative *Staphylococcus* | 5 | |
| *Enterococcus* | 5 | |
| Anaerobic gram-positive bacilli | 2 | |
| Alpha and gamma hemolytic *Streptococcus* | 1 | |
| GBS | 2 | |
| Diphtheroids | 1 | |
| No. of cultures in which co-isolates with *G. vaginalis* and coagulase-negative *Staphylococcus* | | 13 |
| Anaerobic bacteria | 3 | |
| GBS | 3 | |
| *Enterobacteriaceae* | 2 | |
| Gamma hemolytic *Streptococcus* | 2 | |
| Diphtheroids | 2 | |
| *Lactobacillus* | 1 | |
| No. of cultures in which co-isolates with *G. vaginalis* and group D *Enterococcus* | | 16 |
| *Enterobacteriaceae* | 6 | |
| GBS | 5 | |
| Anaerobic bacteria | 2 | |
| Diphtheroids | 2 | |
| Alpha *streptococcus* | 1 | |
| No. of cultres in which a co-isolate is identified with *G. vaginalis*, coagulase-negative *Staphylococcus*, and *Enterococcus* | | 31 |
| Anaerobes | 9 | |
| *Enterobacteriaceae* | 6 | |
| Diphtheroids | 4 | |
| GBS | 3 | |
| *S. aureus* | 3 | |
| Anaerobic gram-positive bacilli | 3 | |
| Alpha and gamma hemolytic *Streptococcus* | 2 | |
| *Mobiluncus* | 1 | |

TABLE 6. Interrelationships: *Staphylococcus*

| |
|---|
| Co-isolation of *S. aureus* and coagulase-negative staphylococci occurred in only 2 of 143 cultures |
| In these 2 cases, multiple isolates including *G. vaginalis*, coagulase-negative *Staphylococcus*, and group D *Enterococcus* were concomitantly present |
| When coagulase-negative *Staphylococcus* was absent, the isolation of *S. aureus* was associated with co-isolation of GBS in 8 of 11 cases |

lates, the compatibility profiling co-isolates with *G. vaginalis* and coagulase-negative *Staphylococcus* were anaerobic bacteria (6), group D *Enterococcus* (5), alpha or gamma *Streptococcus* (3), GBS (2), aerobic gram-positive bacilli (1), and *Enterobacteriaceae* (1). On level three with 4 isolates per culture, compatibility profiling co-isolates with *G. vaginalis*, coagulase-negative *Staphylococcus*, and group D *Enterococcus* (31) were anaerobes (9), *Enterobacteriaceae* (6), diphtheroids (4), GBS (3), *S. aureus* (3), aerobic gram-positive bacilli (3), alpha or gamma *Streptococcus* (2), and *Mobiluncus* (1).

### Inferred Bacterial Interrelationship
### *S. aureus*

Only 7.7% of all *Staphylococcus* were identified as being *S. aureus*. Co-isolation of *S. aureus* and coagulase-negative *Staphylococcus* occurred in only 2 of 132 cultures (Table 6). In these 2 cases, *G. vaginalis*, coagulase-negative *Staphylococcus*, and group D *Enterococcus* were concomitantly isolated along with other isolates indicative of polymicrobial aerobic/anaerobic interaction. When a coagulase-negative *Staphylococcus* was absent, the isolation of *S. aureus* was associated with co-isolation of GBS in 8 of 11 cases. When a coagulase-negative *Staphylococcus* was present, the probability of recovering *S. aureus* was diminished ($P > 0.0001$).

### *GBS*

Of the 49 GBS isolates, 25 had concomitant isolation of one or more of the *Enterobacteriaceae*. Of the 3 cultures which contained either a group F (2) or group G (1) beta hemolytic *Streptococcus*, an *Enterobacteriaceae* was isolated in all 3 instances. In no case was GBS co-isolated with another beta hemolytic *Streptococcus*. If *Lactobacillus* was present, *Gardnerella* was recovered in conjunction with GBS in only 2 of 22 cases; when *Lactobacillus* was absent, *G. vaginalis* was a co-isolate with GBS in 20 of 28 cases ($P < 0.001$). The concomitant isolations of GBS/coagulase-negative *Staphylococcus* and GBS/*S. aureus* were 18 of 132 and 8 of 11, respectively. The increased probability of recovering GBS when *S. aureus* was present as opposed to the coagulase-negative staphylococci was statistically highly significant ($P < 0.0020$). The impact of additive bacteria on GBS is illustrated in Table 7. With addition of compatible dominant co-isolates, the incidence of GBS isolation fell.

TABLE 7. Impact of additive bacteria on GBS

| Co-isolated organisms | No. of cultures with GBS | % of cultures with GBS |
|---|---|---|
| *Lactobacillus* | 20/113 | 17 |
| *Lactobacillus* and coagulase-negative *Staphylococcus* | 7/64 | 11 |
| *Lactobacillus*, coagulase-negative *Staphylococcus*, and *Enterococcus* | 1/26 | 4 |

### *Enterobacteriaceae*

A statistically significant correlation exists between *Enterobacteriaceae* and GBS. Regulation among the *Enterobacteriaceae* is achieved primarily through the elaboration of bacteriocins. Recovery of a single species of the *Enterobacteriaceae* tended to be the rule. Among the 52 isolates of *Escherichia coli*, 6 cultures had another *Enterobacteriaceae* [*Klebsiella pneumoniae* (3), *Proteus mirabilis* (3), *Haemophilus parainfluenzae* (2)]. Of the 10 *Klebsiella* isolates, 8 were *K. pneumoniae* and 2 were *K. oxytoca*. *K. pneumoniae* and *E. coli* were co-isolates in 2 cases. Of the 5 *P. mirabilis* isolates, 3 had co-isolates: 2 *E. coli* and 1 *Acinetobacter*. Multiple *Enterobacteriaceae* isolates occurred in the context of multibacterial (>5) cultures in 3 of the 5 cases.

### *Coagulase-negative Staphylococcus*

Of the 132 patients from whom a *Staphylococcus* was isolated, there were only 2 instances of co-isolation of a coagulase-negative *Staphylococcus* and another *Staphylococcus* species. The presence of a coagulase-negative *Staphylococcus* did not appear to inhibit anaerobic staphylococci (*Peptococcus magnus* and *P. asaccharolyticus;* currently classified with the *Peptostreptococcus*).

### DISCUSSION

The importance of quantitative microbiological data could not be addressed in this study owing to cost considerations. Nevertheless, some insight can be inferred as to the role of the magnitude of cfu/g of vaginal fluid in bacterial dominance.[5] *In vitro* studies of bacterial interference have shown that inhibition is related to numerical dominance. When multiple anaerobes were isolated and/or the total number of isolates from a given vaginal specimen exceeded 5, this combination was presumed to indicate the presence of the anaerobic progression. When the anaerobic progression is functioning, the regulatory influence of traditional dominant aerobes tends to be regulated to a minor role (Monif, unpublished data). When quantitative analysis is done in the anaerobic progression, the number of anaerobes per gram of vaginal fluid exceeded that of aerobic bacteria.[4] Data supporting this thesis are inferred by the *in vitro* inhibition studies done with the *Lactobacillus*/*G. vaginalis* and coagulase-negative *Staphylococcus*/*S. aureus* couplings. When numerical disruption is inferred by the presence of anaerobic progression, the prior *in vitro* demonstrated ability of given aerobic bacteria to inhibit an otherwise susceptible bacteria in the coupling is significantly diminished.

Lactobacilli produce lactic acid, $H_2O_2$, and other antimicrobial metabolics which *in vitro* inhibit other bacteria.[6,7,9] The role of hydrogen peroxide production by the lactobacilli isolates was not addressed in this study. Hydrogen peroxide producing lactobacilli were identified as being present in 90% of women with no vaginal symptomatology.[9] Four percent of cultures contained *Lactobacillus* species that did not produce hydrogen peroxide.[9] Since only lactobacillis which grow on both aerobic and anaerobic media were identified as lactobacilli, it can be presumed that these represent primarily hydrogen peroxide producing isolates. In the experience of Eschenbach et al.,[9] the majority of non-hydrogen peroxide producing lactobacilli grew anaerobically. Klebanoff et al.[7] demonstrated *in vitro* the ability of hydrogen peroxide generating *L. acidophilus* to inhibit *G. vaginalis* and *Bacteroides bivius*. Other investigations have attributed *in vitro* growth inhibition of *G. vaginalis* and other anaerboic bacteria to acid production by lactobacilli.[8] Selected *Enterobacteriaceae*, diphtheroids, and *S. aureus* are catalase positive and theoretically could destroy hydrogen peroxide. Only the presence of diphtheroids appeared to alter the probability of isolation of aerobic lactobacilli.

The *in vivo* data in this study confirm prior *in vitro* data which documented the ability of *Lactobacillus* to inhibit *G. vaginalis*.[1,7,8] When *Lactobacillus* was present, *G. vaginalis* was in concomitant isolates in 7 of 113 cultures. When no anaerobes were present, only one concomitant isolate of *G. vaginalis* was recorded. The other 6 cases occurred in the presence of isolation patterns consistent with the anaerobic progression. The alternate thesis is that these isolates are non-hydrogen producing isolates.

Chaisilwattana and Monif[5] published the only extensive *in vitro* study on the ability of the GBS to inhibit gram-positive and gram-variable aerobic bacterial constituents of the female genital tract. Given numerical dominance created by the *in vitro* techniques used, the GBS inhibit group A, B, C, and G streptococci, lactobacilli, *G. vaginalis*, and most diphtheroid strains. While GBS did not inhibit coagulase-negative staphylococci, *S. aureus*, and most enterococci, GBS isolates were uniformly inhibited by coagulase-negative staphylococci, but were not inhibited by *S. aureus*.

The failure of *in vitro* data concerning GBS/lactobacilli and *G. vaginalis* to be predictive of *in vivo* observations is presumed to be a function of the test technique. The *in vitro* condition necessarily demonstrates that inhibition cannot be duplicated *in vivo*. The demonstrated ability of *Lactobacillus* and *G. vaginalis* for governance makes it unlikely that the numerical discrepancy with GBS required for inhibition could ever be achieved. The *in vivo* absence of other beta hemolytic streptococci or diphtheroids except when evidence of the anaerobic progression was present, is consistent with the prior *in vitro* observations. GBS was recovered in conjunction with both coagulase-negative staphylococci and *S. aureus*. These rates of recovery were statistically different ($P < 0.05$). In the absence of quantitative data, failure of projected inhibition of GBS by coagulase-negative staphylococci is difficult to interpret, whereas the positive correlation of GBS with *S. aureus* is totally consistent with the *in vitro* data. *In vitro*, the coagulase-negative *Staphylococcus* suppressed *S. aureus*. Co-isolation of a coagulase-negative *Staphylococcus* and *S. aureus* did not occur unless polymicrobial bacterial isolates were concomitantly present.

Until quantitative and qualitative studies are concomitantly done within qualitative studies, conjecture-based understanding of the interrelationships within the bacterial flora of the female genital tract will be a combination of *in vitro* and *in vivo* studies, fragmentary quantitative or qualitative data. The number of observations required and the cost of doing such a study properly make it unlikely such data will be forthcoming in the foreseeable future. Further utilization of bacterial compatibility profiling and selective use of quantitative bacteriology will either sustain or refute the concepts advanced in this paper.

### REFERENCES

1. Redondo-Lpez V, Cook RL, Sobel JD: Emergence of lactobacilli in the control and maintenance of the vaginal bacterial microflora. Rev Infect Dis 12:856–872, 1990.
2. Kanler O, Weiss N: Genus *Lactobacillus.* In Sneeth P (ed): Berbey's Manual of Systematic Bacteriology. Vol 2. Baltimore: Williams & Williams, pp 1209–1234, 1986.
3. Gorbach SL, Menda KB, Thadepalli H, Keith L: Anaerobic microflora of the cervix in healthy women. Am J Obstet Gynecol 117:1053–1058, 1973.
4. Bartlett JC, Onderdork AB, Drude E: Quantitative microbiology of the vaginal flora. J Infect Dis 3:535–541, 1981.
5. Chaisilwattana P, Monif GRG: In vitro ability of the group B streptococci to inhibit gram positive and gram variable constituents of the bacterial flora of the female genital tract. Infect Dis Obstet Gynecol 3:91–97, 1995.
6. Hillier SL, Krohn MA, Klebanoff ST, Eschenbach DA: The relationship of hydrogen peroxide producing lactobacilli to bacterial vaginosis and genital microflora in pregnant women. Obstet Gynecol 79:369–373, 1992.
7. Klebanoff SJ, Hillier SL, Eschenbach DA, Waltersdorph AM: Control of the microbial flora of the vagina by $H_2O_2$-generating lactobacilli. J Infect Dis 164:94–100, 1991.
8. Skarin A, Sylwan J: Vaginal lactobacilli inhibit growth of *Gardnerella vaginalis, Mobiluncus* and other bacterial species cultured from vaginal content of women with bacterial vaginosis. Acta Pathol Microbiol Immunol Scand 94:399–403, 1986.
9. Eschenbach DA, Davic PR, Williams B, et al.: Prevalence of hydrogen peroxide producing *Lactobacillus* species in normal women and women with bacterial vaginosis. J Clin Microbiol 27:251–256, 1989.
